# Differential gene expression in response to *Fusarium oxysporum* infection in resistant and susceptible genotypes of flax (*Linum usitatissimum* L.)

**DOI:** 10.1186/s12870-017-1192-2

**Published:** 2017-12-28

**Authors:** Alexey A. Dmitriev, George S. Krasnov, Tatiana A. Rozhmina, Roman O. Novakovskiy, Anastasiya V. Snezhkina, Maria S. Fedorova, Olga Yu. Yurkevich, Olga V. Muravenko, Nadezhda L. Bolsheva, Anna V. Kudryavtseva, Nataliya V. Melnikova

**Affiliations:** 1 Engelhardt Institute of Molecular Biology, Russian Academy of Sciences, Moscow, Russia; 2 All-Russian Research Institute for Flax, Torzhok, Russia

**Keywords:** *Linum usitatissimum*, Flax, Biotic stress, *Fusarium oxysporum*, High-throughput sequencing, ROS, 1,3-beta-glucanase, Cell wall

## Abstract

**Background:**

Flax (*Linum usitatissimum* L.) is a crop plant used for fiber and oil production. Although potentially high-yielding flax varieties have been developed, environmental stresses markedly decrease flax production. Among biotic stresses, *Fusarium oxysporum* f. sp. *lini* is recognized as one of the most devastating flax pathogens. It causes wilt disease that is one of the major limiting factors for flax production worldwide. Breeding and cultivation of flax varieties resistant to *F. oxysporum* is the most effective method for controlling wilt disease. Although the mechanisms of flax response to *Fusarium* have been actively studied, data on the plant response to infection and resistance gene candidates are currently very limited.

**Results:**

The transcriptomes of two resistant and two susceptible flax cultivars with respect to Fusarium wilt, as well as two resistant BC_2_F_5_ populations, which were grown under control conditions or inoculated with *F. oxysporum*, were sequenced using the Illumina platform. Genes showing changes in expression under *F. oxysporum* infection were identified in both resistant and susceptible flax genotypes. We observed the predominant overexpression of numerous genes that are involved in defense response. This was more pronounced in resistant cultivars. In susceptible cultivars, significant downregulation of genes involved in cell wall organization or biogenesis was observed in response to *F. oxysporum*. In the resistant genotypes, upregulation of genes related to NAD(P)H oxidase activity was detected. Upregulation of a number of genes, including that encoding beta-1,3-glucanase, was significantly greater in the cultivars and BC_2_F_5_ populations resistant to Fusarium wilt than in susceptible cultivars in response to *F. oxysporum* infection.

**Conclusions:**

Using high-throughput sequencing, we identified genes involved in the early defense response of *L. usitatissimum* against the fungus *F. oxysporum*. In response to *F. oxysporum* infection, we detected changes in the expression of pathogenesis-related protein-encoding genes and genes involved in ROS production or related to cell wall biogenesis. Furthermore, we identified genes that were upregulated specifically in flax genotypes resistant to Fusarium wilt. We suggest that the identified genes in resistant cultivars and BC_2_F_5_ populations showing induced expression in response to *F. oxysporum* infection are the most promising resistance gene candidates.

**Electronic supplementary material:**

The online version of this article (10.1186/s12870-017-1192-2) contains supplementary material, which is available to authorized users.

## Background

Flax (*Linum usitatissimum* L.) is a widely distributed crop, which is used for fiber and oil production [1]. Genetic polymorphism of *L. usitatissimum* and related species is well characterized [2–7] and could be used for the breeding of improved cultivars. Although potentially high-yielding flax varieties have previously been developed, biotic and abiotic stresses can markedly decrease flax production. Therefore, the molecular mechanisms underlying the responses of flax to unfavorable environments are intensively studied. In this regard, changes in the expression of stress-responsive genes and microRNAs have been detected in flax plants under abiotic stresses, such as drought [8], salinity and alkalinity [9, 10], nutrient imbalance [11], and high concentrations of aluminum ions [12, 13].

Among biotic stresses, *Fusarium oxysporum* f. sp. *lini* is recognized as one of the most devastating flax pathogen. It causes wilt disease, which is one of the major limiting factors for flax production in most of the flax-growing areas worldwide. Epidemics of the disease can result in an 80% to 100% loss in yield [14]. Breeding and cultivation of flax varieties resistant to *F. oxysporum* is the most effective method for controlling wilt disease, and in this regard, evaluation of flax germplasm for resistance to Fusarium wilt has revealed accessions with potential utility in breeding programs [15]. Furthermore, the search for genes conferring resistance to *Fusarium* infection is currently underway, and amplified fragment length polymorphism (AFLP) analysis of a flax mapping population derived from doubled haploid lines has already led to the identification of two quantitative trait loci associated with resistance to Fusarium wilt [16]. However, the genes that define resistance to *Fusarium* in some flax genotypes remain unknown.

Alterations that occur in flax plants under *Fusarium* infection have been actively studied and, in some cases, the molecular mechanisms underlying responses have been elucidated. The role of pathogenesis-related (PR) proteins, including chitinase and β-1,3-glucanase, in response to *Fusarium* has been revealed. Upregulation of chitinase genes has been identified in flax plants under *F. oxysporum* infection [17]. Flax lines with ectopic expression of the β-1,3-glucanase gene or overexpression of endogenous β-1,3-glucanase gene show enhanced resistance to *F. oxysporum* and *F. culmorum* [18, 19]. Moreover, those flax plants with overexpressed β-1,3-glucanase have increased contents of antioxidants, phenolics, and polyamines, as well as alterations in cell wall biopolymer composition [18–20]. Enhanced resistance via an increase in antioxidant activity has also been observed in transgenic flax plants with increased contents of flavonoids, carotenoids, or other terpenoids [21–23]. Furthermore, the involvement of antioxidants and cell wall components in the flax response to *Fusarium* has been demonstrated in different plant material, including cell cultures, seeds, and seedlings. Oxidative burst, activation of lipid peroxidation, and phenylpropanoid metabolism have been observed in flax cells under interaction with *F. oxysporum* [24]. The contribution of the antioxidant potential of phenylpropanoids, which accumulate in seeds, and pectin content in flax resistance to *Fusarium* have also been identified [25], as have the changes in pectin metabolism in flax seedlings under *Fusarium* infection [26]. Changes in the expression of genes participating in stress response, defense response, metabolism regulation, and, in particular, the phenylpropanoid pathway have been detected in flax plants during the early stages of *Fusarium* infection [27], and it has been suggested that an increase in methyl salicylate level in flax plants in response to *F. oxysporum* is associated with activation of the phenylpropanoid pathway [28]. The role of polyamines in response to *Fusarium* has also been revealed [29]. RNA-seq of flax plants after infection with *F. oxysporum* allowed identification of changes in the expression of genes involved in signal transduction, regulation of transcription, hormone signaling, reactive oxygen species (ROS) regulation, secondary metabolism, and other processes [17]. Thus, it has been variously established that PR-proteins, antioxidants, and cell wall components are involved in the flax response to *Fusarium* infection.

In the present study, we used high-throughput sequencing of transcriptomes to evaluate the changes in flax gene expression under *F. oxysporum* infection in resistant and susceptible flax cultivars and BC_2_F_5_ populations, the latter of which were obtained from crosses between resistant and susceptible flax cultivars and then selected for both resistance to *F. oxysporum* and phenotypical similarity with the susceptible parent for several generations. This approach allowed us to identify candidate genes conferring resistance to *F. oxysporum* infection in *L. usitatissimum.*

## Methods

### Plant material

Experiments for identification of flax cultivars with resistance and susceptibility to *F. oxysporum* have previously been performed at the All-Russian Research Institute for Flax (Torzhok, Russia). Based on the obtained results, two resistant (Dakota and #3896) and two susceptible (AP5 and TOST) cultivars were selected for examination in the present study. In addition, hybrids of cultivars resistant and susceptible to *F. oxysporum* were obtained at the same institute. The susceptible cultivar AP5 was crossed with both resistant cultivars (Dakota and #3896), and the resulting F_1_ plants were backcrossed to AP5. Subsequently, selection against a provocative background (soil inoculated with an isolate of *Fusarium oxysporum* f. sp. *lini*) was performed and plants that were resistant but phenotypically similar to the AP5 cultivar were selected. A further backcross was then conducted and resistant plants similar to AP5 were again selected. Thereafter, self-pollination of BC_2_F_1_ individuals and selection of resistant families that were phenotypically similar to AP5 were performed for five generations. As a result, BC_2_F_5_ populations resistant to *F. oxysporum* were obtained: #3896 × АР5 (recurrent parent АР5) and Dakota × АР5 (recurrent parent АР5).

Thus, two cultivars with resistance (Dakota and #3896) and two cultivars with susceptibility (AP5 and TOST) to *F. oxysporum,* as well as resistant BC_2_F_5_ populations (#3896 × АР5 and Dakota × АР5), were used in our study. Seeds were initially sterilized in 70% ethanol for 1 min and in 1% sodium hypochlorite for 20 min, and then rinsed 15 times in sterile deionized water. The plants were grown in sterile 16 mm × 150 mm glass tubes on Murashige-Skoog medium in a growth chamber at 22 °C with a 16 h day and 8 h night.

*Fusarium oxysporum* f. sp. *lini* pathogenic isolate #39 from the phytopathogen collection of the All-Russian Research Institute for Flax was grown on potato dextrose agar for 5 days prior to inoculation. This was the same isolate that was applied for selection of resistant families after crosses between Dakota and AP5 and #3896 and AP5. Seven-day-old flax plants were inoculated with 1 ml of a 10^5^ per ml preparation of *F. oxysporum* spores (fungal infection) or with 1 ml of sterile water (control). After 48 h, when necrosis of roots had appeared, root tips (approx. 5 mm in length), in which the infection initially occurred, were collected and frozen in liquid nitrogen. It was previously shown that *F. oxysporum* displays a clear preference for the root tips of flax. Two days after inoculation, the fungus was mainly distributed around the root tips, with significantly less presence in the elongation zone and in lateral branches [30]. Accordingly, for studying the early infection stages, we selected root tips as the most preferable experimental material. In total, approximately 120 infected plants and 120 plants grown under control conditions were obtained for four cultivars and two BC_2_F_5_ populations.

### Library preparation and transcriptome sequencing

Total RNA was extracted from pooled plant samples using an RNeasy Plant Mini Kit (Qiagen, USA). Each pool included 10–12 plants of each cultivar/population under control conditions or fungal infection. Thus, 24 RNA samples were extracted in two replicates under either fungal infection or control conditions: Dakota, #3896, AP5, TOST, BC_2_F_5_ #3896 × АР5, and BC_2_F_5_ Dakota × АР5. RNA concentration and quality were evaluated using a Qubit 2.0 fluorometer (Life Technologies, USA) and Agilent 2100 Bioanalyzer (Agilent Technologies, USA). High-quality RNA samples (RNA integrity number not less than 8.0) were used for cDNA library preparation with polyA-based mRNA capture using a TruSeq Stranded Total RNA Library Prep Kit (Illumina, USA). The quality of the 24 obtained libraries was evaluated using the Agilent 2100 Bioanalyzer. The libraries were sequenced using a NextSeq 500 high-throughput sequencer (Illumina) and paired-end reads (80 + 80 nucleotides) were obtained.

### High-throughput sequencing data analysis

Illumina reads were trimmed and filtered using Trimmomatic [31] and then *F. oxysporum* reads were filtered out by mapping to the *F. oxysporum* reference genome and transcriptome (NCBI assembly/WGS identifier ASM14995v2/AAXH01) using bowtie2. The remaining reads were used for transcriptome assembly using Trinity 2.4.0 with the default parameters [32]. The assembly was performed (1) for each cultivar/population, (2) for each BC_2_F_5_ population jointly with the corresponding parents, and (3) for all sequenced flax samples together.

The quality of assemblies was assessed with N50, ExN50, and L50 statistics using QUAST 4.5 and Trinity utilities. Trasncripts that were less than 200 nucleotides in length were excluded from subsequent analysis. The transcripts were also analyzed with BUSCO to evaluate the completeness of the assembly [33]. Trinotate pipeline was then used for the annotation of the assembled transcripts (http://trinotate.github.io/). The derived transcripts were analyzed for the presence of open reading frames (ORFs) using TransDecoder [34]. The transcripts and the predicted proteins were aligned to the UniProt database using blastx and blastp, respectively. The protein sequences were scanned for the presence of PFAM domains using HMMER [35, 36]. On the basis of these data, a local SQLite database was constructed and transferred to Trinotate. Finally, the transcripts were annotated using the Gene Ontology (GO), KEGG, and COG databases.

Then reads were mapped to the assembled transcripts (all nine assemblies) and quantified using bowtie2 [37] and rsem [38]. Read counts per transcript and per gene were calculated. The derived read count data were analyzed using edgeR [39]. After normalization using the TMM method, we attempted to identify the following gene responses:

1. up- and down-regulated in flax genotypes resistant to *F. oxysporum* (Dakota, #3896, #3896 × AP5, Dakota × AP5) in response to *F. oxysporum* infection;
2. up- and down-regulated in flax genotypes susceptible to *F. oxysporum* (AP5 and TOST) in response to *F. oxysporum* infection;
3. induced in response to *F. oxysporum* infection in the resistant cultivars and BC_2_F_5_ populations, but not (or less so) in the susceptible cultivars.

Only genes with a *CPM* greater than 2.0 for at least three samples were used for further analysis. The *t-*test was used to determine *p*-values. False discovery rate (FDR) values were derived using the Benjamini-Hochberg p-value adjustment procedure. The gene set enrichment analysis (GSEA) with GO data was performed using Goseq [40]. For this analysis, we used lists of the top 50, 100, 200, 500, 1000, and 2000 upregulated or downregulated genes, separately. Different GO terms were enriched when we used distinct top differentially expressed gene list sizes.

## Results

### High-throughput sequencing of flax plants

We sequenced the transcriptomes of flax cultivars showing resistance (Dakota and #3896) and susceptibility (AP5 and TOST) to Fusarium wilt, which were exposed to control conditions or inoculated with *F. oxysporum*. We also sequenced the transcriptomes of BC_2_F_5_ populations (#3896 × АР5, recurrent parent АР5; Dakota × АР5, recurrent parent АР5) with resistance to *F. oxysporum.* From 45.7 to 55.7 million reads were generated for each cultivar or BC_2_F_5_ population under control conditions or *F. oxysporum* infection. For plants inoculated with pathogen samples, 30%–46% of reads were mapped to the *F. oxysporum* genome and transcriptome. In control plants, less than 0.2% of reads were mapped to the *F. oxysporum* sequences (the most of these reads were rRNA-originated), which can be explained by the similarity of some flax and *Fusarium* sequences. After filtering against the *F. oxysporum* genome and transcriptome, the following transcriptome assemblies were performed: (1) for each cultivar and BC_2_F_5_ population separately; (2) for each BC_2_F_5_ population jointly with the corresponding parents; (3) for all cultivars and BC_2_F_5_ populations together. Approximately 107–111 thousand transcripts related to 52–55 thousand genes were derived for each cultivar and BC_2_F_5_ population. For the BC_2_F_5_ population combined with the corresponding parents and for all analyzed samples, a larger number of genes and transcripts were identified (Table 1). In the annotation of transcripts, approximately 50% of the transcripts were successfully mapped to UniProt using blastx. For almost 60% of the transcripts, long ORFs were identified. Approximately 23,000 transcripts passed the *CPM* threshold and were used for differential expression analysis. To assess the completeness of transcriptome assemblies, we mapped the transcripts to the database of single-copy orthologs among Embryophyta with BUSCO. The assembly that was derived with the complete pool of reads (all samples), as well as the assemblies that included either susceptible or resistant cultivars and populations demonstrated equally good completeness: 91%–92% of complete orthologs were present. The assemblies that included only one cultivar/population revealed slightly lower values (85%–88%).

**Table 1:** Transcriptome assembly statistics for flax cultivars and BC_2_F_5_ populations

Feature	АР5	TOST	Dakota	#3896	Dakota × АР5	#3896 × АР5	Dakota × АР5, Dakota, АР5	#3896 × АР5, #3896,АР5	All samples
Genes	52,659	52,802	52,287	55,062	51,758	52,088	72,128	73,473	89,290
Transcripts	110,764	108,737	107,255	111,051	108,107	109,009	151,218	154,343	183,905
GC-content	46	46	46	46	46	46	46	46	46
N50	1776	1804	1806	1810	1810	1831	1874	1859	1882
Median contig length	998	1000	1010	987	1015	1032	997	987	923
Average contig length	1212	1220	1225	1214	1228	1245	1247	1235	1216
Total assembled bases, Mb	134.2	132.7	131.3	134.8	132.7	135.8	188.5	190.7	223.7

### Changes in gene expression in flax plants under *F. Oxysporum* infection

Expression analysis was performed for identification of up- and down-regulated genes under *F. oxysporum* infection. Expression levels of identified transcripts were evaluated for all received assemblies under control conditions and at 48 h post-inoculation in the following groups: (1) separately for each of the studied cultivars and BC_2_F_5_ populations; (2) pool of cultivars resistant (Dakota and #3896) and pool of cultivars susceptible (AP5 and TOST) to Fusarium wilt; (3) pools of resistant cultivars and derived BC_2_F_5_ populations (Dakota and Dakota × АР5; #3896 and #3896 × АР5); (4) pool of resistant cultivars and BC_2_F_5_ populations (Dakota, #3896, Dakota × АР5, and #3896 × АР5). For all assembly variants, the top differentially expressed genes were mostly similar. Further, the results of expression analysis for the assembly from all samples are presented.

Genes showing changes in expression under *F. oxysporum* infection were identified in both resistant and susceptible flax genotypes. GO analysis was performed for the top 100 up- and down-regulated genes. In the flax genotypes with resistance to *F. oxysporum* (resistant cultivars and BC_2_F_5_ populations), the top 100 upregulated genes were related to NAD(P)H dehydrogenase activity, oxidoreductase activity, respiratory chain complex I, and mitochondrial parts (Table 2). In the susceptible cultivars, the top 100 upregulated genes were related to other categories, including translation, ribosome, biosynthetic process, and cytosolic part (Table 3). GO analysis of the top 100 downregulated genes also revealed differences between resistant and susceptible genotypes: in resistant genotypes, the genes were related to microtubule, kinesin complex, cytoskeletal part, cell junction, clathrin coat, and adenyl nucleotide binding (Table 4); in susceptible genotypes, the genes were related to cell wall, external encapsulating structure, transferase activity, transferring glycosyl groups, polysaccharide metabolic process, water channel activity, intrinsic component of membrane, and xyloglucan metabolic process (Table 5).

**Table 2:** Gene ontology analysis for the top 100 upregulated genes in flax genotypes resistant to *Fusarium oxysporum* (Dakota, #3896, #3896 × AP5, and Dakota × AP5)

GO category	Term	Observed number of genes	Expected number of genes	*p-value*	*FDR*
GO:0008137	NADH dehydrogenase (ubiquinone) activity	6	0.15	1.5E-08	1.1E-04
GO:0050136	NADH dehydrogenase (quinone) activity	6	0.15	1.5E-08	1.1E-04
GO:0003954	NADH dehydrogenase activity	6	0.17	3.3E-08	1.7E-04
GO:1,990,204	oxidoreductase complex	7	0.38	5.0E-08	1.9E-04
GO:0005747	mitochondrial respiratory chain complex I	5	0.14	1.2E-07	2.8E-04
GO:0045271	respiratory chain complex I	5	0.14	1.2E-07	2.8E-04
GO:0030964	NADH dehydrogenase complex	5	0.14	1.4E-07	2.8E-04
GO:0016655	oxidoreductase activity, acting on NAD(P)H, quinone or similar compound as acceptor	6	0.23	1.5E-07	2.8E-04
GO:0044455	mitochondrial membrane part	8	0.61	2.0E-07	3.4E-04
GO:0044429	mitochondrial part	14	2.58	4.0E-07	6.2E-04
GO:0016651	oxidoreductase activity, acting on NAD(P)H	7	0.56	1.5E-06	2.1E-03
GO:0009435	NAD biosynthetic process	3	0.04	2.8E-05	3.6E-02
GO:1,901,566	organonitrogen compound biosynthetic process	12	2.93	4.4E-05	5.3E-02

*Note:* One gene can belong to more than one GO category. FDR – false discovery rate

**Table 3:** Gene ontology analysis for the top 100 upregulated genes in the flax genotypes susceptible to *Fusarium oxysporum* (AP5 and TOST)

GO category	Term	Observed number of genes	Expected number of genes	*p-value*	*FDR*
GO:0002181	cytoplasmic translation	12	0.24	0	0
GO:0003735	structural constituent of ribosome	30	1.61	1.6E-32	1.3E-28
GO:0006412	translation	29	1.87	1.9E-28	9.8E-25
GO:0005198	structural molecule activity	30	2.30	1.3E-27	5.0E-24
GO:0044391	ribosomal subunit	21	1.08	8.8E-23	2.7E-19
GO:0030529	intracellular ribonucleoprotein complex	34	4.47	1.8E-22	4.7E-19
GO:0044445	cytosolic part	21	1.26	2.4E-21	5.3E-18
GO:0022625	cytosolic large ribosomal subunit	14	0.46	1.5E-18	2.8E-15
GO:0015934	large ribosomal subunit	14	0.61	2.3E-16	3.9E-13
GO:0009059	macromolecule biosynthetic process	41	11.92	5.1E-14	8.0E-11
GO:0034645	cellular macromolecule biosynthetic process	39	11.27	2.2E-13	3.1E-10
GO:1,901,576	organic substance biosynthetic process	49	19.57	4.9E-12	6.3E-09
GO:0009058	biosynthetic process	49	20.57	3.0E-11	3.6E-08
GO:0044249	cellular biosynthetic process	47	19.05	3.9E-11	4.4E-08
GO:0032991	macromolecular complex	41	15.18	2.4E-10	2.5E-07
GO:0022627	cytosolic small ribosomal subunit	7	0.32	5.9E-09	5.7E-06
GO:0005840	ribosome	11	1.28	1.1E-08	1.0E-05
GO:0044267	cellular protein metabolic process	29	9.71	4.8E-08	4.2E-05
GO:0015935	small ribosomal subunit	7	0.47	2.0E-07	1.6E-04
GO:0019538	protein metabolic process	30	11.46	5.0E-07	3.9E-04
GO:0006414	translational elongation	4	0.07	8.9E-07	6.6E-04
GO:0045460	sterigmatocystin metabolic process	4	0.13	1.2E-05	8.2E-03
GO:0045461	sterigmatocystin biosynthetic process	4	0.13	1.2E-05	8.2E-03
GO:1,901,378	organic heteropentacyclic compound biosynthetic process	4	0.18	3.5E-05	2.3E-02
GO:1,901,376	organic heteropentacyclic compound metabolic process	4	0.18	3.7E-05	2.3E-02
GO:0009403	toxin biosynthetic process	4	0.21	6.6E-05	3.9E-02
GO:0070069	cytochrome complex	3	0.10	6.8E-05	3.9E-02

*Note:* One gene can belong to several GO categories. FDR – false discovery rate

**Table 4:** Gene ontology analysis for the top 100 downregulated genes in the flax genotypes resistant to *Fusarium oxysporum* (Dakota, #3896, #3896 × AP5, Dakota × AP5)

GO category	Term	Observed number of genes	Expected number of genes	*p-value*	*FDR*
GO:0003777	microtubule motor activity	10	0.35	0	0
GO:0007018	microtubule-based movement	11	0.40	0	0
GO:0007017	microtubule-based process	15	1.15	1.2E-12	5.5E-09
GO:0005874	microtubule	15	1.16	1.4E-12	5.5E-09
GO:0005871	kinesin complex	9	0.30	4.8E-11	1.3E-07
GO:0006928	movement of cell or subcellular component	11	0.60	5.0E-11	1.3E-07
GO:0003774	motor activity	10	0.48	1.8E-10	4.0E-07
GO:0005875	microtubule associated complex	9	0.50	5.4E-09	1.1E-05
GO:0009524	phragmoplast	8	0.42	1.8E-08	3.1E-05
GO:0044430	cytoskeletal part	15	2.42	6.7E-08	1.0E-04
GO:0008574	ATP-dependent microtubule motor activity, plus-end-directed	4	0.06	4.4E-07	6.2E-04
GO:0000911	cytokinesis by cell plate formation	5	0.15	9.5E-07	1.2E-03
GO:0009506	plasmodesma	13	3.14	6.2E-06	7.4E-03
GO:0005911	cell-cell junction	13	3.24	9.2E-06	1.0E-02
GO:0030130	clathrin coat of trans-Golgi network vesicle	3	0.06	9.8E-06	1.0E-02
GO:0030054	cell junction	13	3.36	1.5E-05	1.4E-02
GO:0030132	clathrin coat of coated pit	3	0.06	1.7E-05	1.5E-02
GO:0030125	clathrin vesicle coat	3	0.07	2.8E-05	2.4E-02
GO:0030118	clathrin coat	3	0.08	4.9E-05	4.0E-02
GO:0005524	ATP binding	28	13.51	5.9E-05	4.3E-02
GO:0061640	cytoskeleton-dependent cytokinesis	4	0.18	5.9E-05	4.3E-02
GO:0032549	ribonucleoside binding	30	15.15	6.5E-05	4.3E-02
GO:1,902,410	mitotic cytokinetic process	5	0.35	6.6E-05	4.3E-02
GO:0001882	nucleoside binding	30	15.17	6.6E-05	4.3E-02
GO:0032559	adenyl ribonucleotide binding	28	13.66	7.1E-05	4.4E-02
GO:0030554	adenyl nucleotide binding	28	13.68	7.3E-05	4.4E-02

*Note:* One gene can belong to more than one GO category. FDR – false discovery rate

**Table 5:** Gene ontology analysis for the top 100 downregulated genes in the flax genotypes susceptible to *Fusarium oxysporum* (AP5 and TOST)

GO category	Term	Observed number of genes	Expected number of genes	*p-value*	*FDR*
GO:0071555	cell wall organization	21	2.32	4.6E-15	7.1E-11
GO:0071554	cell wall organization or biogenesis	22	2.78	1.8E-14	1.0E-10
GO:0045229	external encapsulating structure organization	21	2.52	1.9E-14	1.0E-10
GO:0005576	extracellular region	26	5.10	6.7E-13	2.6E-09
GO:0005618	cell wall	15	2.89	6.5E-08	1.7E-04
GO:0030312	external encapsulating structure	15	2.89	6.5E-08	1.7E-04
GO:0048046	apoplast	11	1.53	1.1E-07	2.4E-04
GO:0016762	xyloglucan:xyloglucosyl transferase activity	4	0.12	3.2E-06	5.1E-03
GO:0031225	anchored component of membrane	8	0.89	3.5E-06	5.1E-03
GO:0016757	transferase activity, transferring glycosyl groups	13	2.81	3.7E-06	5.1E-03
GO:0005976	polysaccharide metabolic process	12	2.53	3.9E-06	5.1E-03
GO:0042546	cell wall biogenesis	7	0.72	4.3E-06	5.1E-03
GO:0005372	water transmembrane transporter activity	4	0.16	4.5E-06	5.1E-03
GO:0015250	water channel activity	4	0.16	4.5E-06	5.1E-03
GO:0031226	intrinsic component of plasma membrane	10	1.82	1.0E-05	1.1E-02
GO:0031224	intrinsic component of membrane	39	20.87	1.8E-05	1.7E-02
GO:0046658	anchored component of plasma membrane	6	0.57	2.5E-05	2.3E-02
GO:0005975	carbohydrate metabolic process	18	6.30	2.9E-05	2.5E-02
GO:0004553	hydrolase activity, hydrolyzing O-glycosyl compounds	10	2.12	3.8E-05	3.1E-02
GO:0009505	plant-type cell wall	7	1.05	4.9E-05	3.7E-02
GO:0010411	xyloglucan metabolic process	4	0.22	4.9E-05	3.7E-02
GO:0030570	pectate lyase activity	3	0.09	6.9E-05	4.7E-02
GO:0016798	hydrolase activity, acting on glycosyl bonds	10	2.29	7.0E-05	4.7E-02

*Note:* One gene can belong to more than one GO category. FDR – false discovery rate

### Genes specifically upregulated in resistant genotypes of flax in response to *F. Oxysporum*

For identification of candidate genes responsible for resistance to *F. oxysporum* in flax, we searched for genes that were up- or down-regulated in resistant cultivars and BC_2_F_5_ populations under *F. oxysporum* infection, but did not show a change in expression (or showed less change) in susceptible cultivars. The full results are presented in Additional file 1 and the results for the top 30 differentially expressed genes (excluding two unknown genes) are presented in Table 6.

**Table 6:** Genes that were specifically changed in resistant genotypes of flax in response to *Fusarium oxysporum* infection

Blast symbol	Blast name	*log*_*2*_ *FC*	*p-value*	*FDR*
*SRG1*	Protein SRG1	5.2	1.0E-12	2.5E-08
*U73C3*	UDP-glycosyltransferase 73C3	3.8	5.5E-11	6.1E-07
*AATP5*; *AATPA*; *ASD*	AAA-ATPase ASD, mitochondrial AAA-ATPase At3g28510 AAA-ATPase At3g28600	2.5	7.7E-11	6.1E-07
*HSP7C*	Heat shock 70 kDa protein 3	−2.1	1.9E-10	9.6E-07
*HSP72*	Heat shock cognate 70 kDa protein 2	−1.9	2.0E-10	9.6E-07
*E13B*	Glucan endo-1,3-beta-glucosidase	4.1	2.6E-10	1.0E-06
*HFA2D*; *HFA7B*	Heat stress transcription factor A-2d Heat stress transcription factor A-7b	−1.9	1.6E-09	4.8E-06
*EP1G*	Epidermis-specific secreted glycoprotein EP1	2.2	2.0E-09	5.1E-06
*CA4*; *CB24*	Chlorophyll a-b binding protein 4, chloroplastic Chlorophyll a-b binding protein P4, chloroplastic	−2.6	2.3E-09	5.1E-06
*EXLB1*	Expansin-like B1	2.7	2.4E-09	5.1E-06
*COL5*	Zinc finger protein CONSTANS-LIKE 5	−2.8	2.7E-09	5.2E-06
*TIP12*	Probable aquaporin TIP1–2	−3.3	3.8E-09	6.9E-06
*HIP3*; *HIP6*	Heavy metal-associated isoprenylated plant protein 3 Heavy metal-associated isoprenylated plant protein 6	1.8	7.2E-09	1.2E-05
*GPAT1*	Glycerol-3-phosphate acyltransferase 1	2.5	8.3E-09	1.3E-05
*MYB36*; *MYB87*	Transcription factor MYB36 Transcription factor MYB87	1.8	1.0E-08	1.5E-05
*ACCH1*	1-aminocyclopropane-1-carboxylate oxidase homolog 1	2.1	1.3E-08	1.9E-05
*AGL62*	Agamous-like MADS-box protein AGL62	4.3	3.2E-08	4.2E-05
*TNG2*	Transport and Golgi organization 2 homolog	1.8	4.6E-08	5.8E-05
*HSP83*	Heat shock protein 83	−1.5	5.3E-08	6.3E-05
*GDPD1*	Glycerophosphodiester phosphodiesterase GDPD1, chloroplastic	2.5	5.6E-08	6.3E-05
*SAU32*; *SAU72*	Auxin-responsive protein SAUR32 Auxin-responsive protein SAUR72	1.7	7.2E-08	7.5E-05
*KSB*	Ent-kaur-16-ene synthase, chloroplastic	3.3	7.3E-08	7.5E-05
*ERD10*; *ERD14*	Dehydrin ERD 10 Dehydrin ERD14	−2.4	8.7E-08	8.6E-05
*RFS*	Galactinol-sucrose galactosyltransferase	−2.0	1.1E-07	1.0E-04
*NAS4*	Probable nicotianamine synthase 4	−2.3	1.1E-07	1.0E-04
*C82C2*; *C82C4*	Cytochrome P450 82C2 Cytochrome P450 82C4	2.7	1.6E-07	1.4E-04
*IQD31*; *STR15*	Protein IQ-DOMAIN 31; Rhodanese-like domain-containing protein 15, chloroplastic	2.6	1.7E-07	1.4E-04
*UGT8*	7-deoxyloganetic acid glucosyltransferase	2.3	2.0E-07	1.5E-04

*Note: FC* – the ratio of average counts per million (*CPM*) in resistant genotypes under *F. oxysporum* infection to the average *CPM* in resistant genotypes under control conditions. FDR – false detection rate

Upregulation was revealed for genes encoding SRG1 (senescence-related gene 1) protein, UDP-glycosyltransferase 73C3 (UGT73C3), AAA-ATPase ASD, mitochondrial (AATPA), glucan endo-1,3-beta-glucosidase, MYB transcription factors, ERD dehydrins, and Auxin-responsive protein SAUR, among others. We suggest that the identified genes with specifically induced expression in response to *F. oxysporum* infection in resistant cultivars and resistant BC_2_F_5_ populations are the most promising resistance gene candidates.

GO terms with the most significant differences between flax genotypes resistant and susceptible to the fungus, and the expression profiles of related genes are presented in Additional file 2. In resistant cultivars and populations, genes involved in the response to biotic stimulus and stress, defense response, antioxidant activity, and cell wall organization or biogenesis were more strongly upregulated than in the susceptible cultivars.

## Discussion

Plant mechanisms of response to *Fusarium* infection include synthesis of PR proteins and antimicrobial compounds, production of ROS, and changes in cell wall structure [41–45]. In the present study, we evaluated the changes in gene expression in response to *F. oxysporum* infection in resistant and susceptible flax cultivars and resistant BC_2_F_5_ populations. The advantage of our study is the use of the two BC_2_F_5_ populations, which were obtained from crosses between the examined resistant and susceptible cultivars, and which are resistant to *F. oxysporum* but phenotypically similar to the susceptible parent. This approach allowed us to compare the changes in gene expression under *F. oxysporum* infection in resistant and susceptible genotypes and to identify genes that were specifically induced in resistant flax plants in response to the infection.

Significant downregulation of genes involved in cell wall organization or biogenesis was detected in response to *F. oxysporum* in susceptible cultivars (AP5 and TOST). However, we observed no similar trend in resistant cultivars and populations. It could be suggested that, in susceptible cultivars, changes in apoplast structure in response to *F. oxysporum* are more pronounced. The role of cell wall compounds in the response of flax [26, 46] and other plant species [47–49] to *F. oxysporum* has been revealed previously. However, the present study is the first to identify the differential expression of genes related to cell wall organization or biogenesis in flax cultivars and BC_2_F_5_ populations with different resistance to Fusarium wilt.

A number of the top 100 upregulated genes in resistant cultivars are related to NAD(P)H oxidase activity. In susceptible cultivars, we also revealed upregulation of NAD(P)H oxidase-related genes; however, most of these were not included in the top 100 upregulated genes. NAD(P)H oxidases are involved in ROS signaling and stress response in plants, and are one of the sources of ROS that are induced in response to pathogen attack and involved in early defense responses via an oxidative burst [45, 50–57]. NADPH oxidase upregulation and early oxidative burst have been revealed in a resistant banana cultivar in response to *F. oxysporum* infection [56, 58, 59]. In flax plants, we observed a similar trend. ROS signaling associated with NAD(P)H oxidases could be one of the mechanisms constituting the *L. usitatissimum* defense response to *F. oxysporum.* We accordingly suggest that NAD(P)H oxidases could be promising candidates for proteins that participate in the defense response against *F. oxysporum* in flax.

**Fig. 1:**
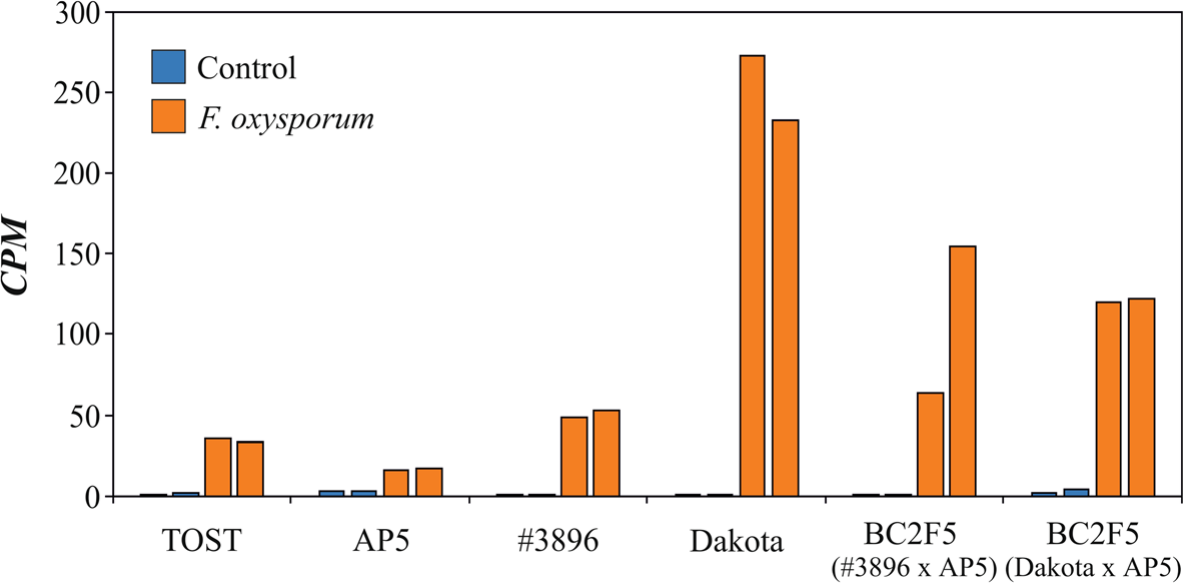
Expression levels of the beta-1-3-glucanase gene in flax genotypes resistant (Dakota, #3896, BC_2_F_5_ #3896 × АР5, BC_2_F_5_ Dakota × АР5) and susceptible (AP5 and TOST) to Fusarium wilt under control conditions and *F. oxysporum* infection. High-throughput sequencing data. *CPM* – count per million. Two biological replicates (10–12 plants in each) are represented for each condition

The genes that were induced more strongly in the resistant genotypes of flax compared with the susceptible genotypes in response to *F. oxysporum* infection are involved in crucial biological processes, including transcription regulation, auxin signaling, stress response, and photosynthesis. The most significant upregulation was observed for genes encoding SRG1 protein, UGT73C3, AATP5, glucan endo-1,3-beta-glucosidase (beta-1-3-glucanase), and epidermis-specific secreted glycoprotein EP1. Among these proteins, beta-1-3-glucanase is the most well-known fungal-responsive protein in flax. This enzyme hydrolyzes beta-1,3-glucans of the cell wall in fungi, and the role of this protein in plant defense against pathogens is well known [60–64]. In flax, increased resistance to *F. oxysporum* and *F. culmorum* has been observed in transgenic flax lines containing the potato beta-1,3-glucanase gene, and in plants overexpressing the beta-1,3-glucanase gene [18, 19]. We also revealed the upregulation of this gene in flax plants in response to *F. oxysporum* infection, and the changes were observed to be stronger in the cultivars and BC_2_F_5_ populations showing resistance to Fusarium wilt (Fig. 1). In resistant genotypes under *F. oxysporum* infection, the induction of beta-1,3-glucanase expression was more pronounced compared with that in susceptible genotypes (*p* < 0.01, Mann–Whitney test). Moreover, under the stress conditions, the expression level of beta-1,3-glucanase was significantly higher in resistant cultivars and populations (*p* < 0.05), whereas under control conditions, there was no significant difference between the resistant and susceptible genotypes.

Thus, in response to *F. oxysporum* infection, we revealed changes in the expression of genes that encode PR proteins, and also in genes that are involved in ROS production and cell wall structure change. Furthermore, we identified genes that were specifically upregulated in flax genotypes resistant to Fusarium wilt. Special attention should be given to these genes in further searches for resistance gene candidates. Our work complements previously obtained results on flax response to *F. oxysporum* infection and provides a basis for detailed investigations of flax defense mechanisms against Fusarium wilt.

## Conclusions

In the present study, we used high-throughput sequencing to search for genes involved in the early defense response of *L. usitatissimum* against infection by the fungus *F. oxysporum*. To this end, we first used resistant and susceptible flax cultivars and *F. oxysporum-*resistant BC_2_F_5_ populations, which were obtained from crosses between the resistant and susceptible flax cultivars. An analysis of gene expression revealed diverse patterns of differentially expressed genes for resistant and susceptible flax genotypes. Genes involved in response to biotic stimulus and stress, defense response, antioxidant activity, and cell wall organization or biogenesis were more strongly upregulated in the resistant genotypes than in the susceptible genotypes. Moreover, we identified genes that were specifically induced in genotypes resistant to Fusarium wilt in response to *F. oxysporum* infection. These genes are the most promising candidates for genes conferring resistance to *F. oxysporum* infection in *L. usitatissimum*.

## Additional files

Additional file 1: Genes for which expression was specifically changed in resistant genotypes of flax in response to *Fusarium oxysporum* infection. (XLSX 9584 kb)

Additional file 2: Expression profiles of genes for selected gene ontology (GO) terms in susceptible and resistant flax genotypes in response to *Fusarium oxysporum* infection. (XLSX 861 kb)
